# Gender specific somatic symptom burden and mortality risk in the general population

**DOI:** 10.1038/s41598-022-18814-4

**Published:** 2022-09-05

**Authors:** Seryan Atasoy, Constanze Hausteiner-Wiehle, Heribert Sattel, Hamimatunnisa Johar, Casper Roenneberg, Annette Peters, Karl-Heinz Ladwig, Peter Henningsen

**Affiliations:** 1 Department of Psychosomatic Medicine and Psychotherapy, Klinikum Rechts Der Isar, University Hospital Rechts Der Isar, Technische Universität München, Langerstr. 3, 81676 Munich, Germany; 2 Department of Psychosomatic Medicine and Psychotherapy, University of Giessen and Marburg, Marburg, Germany; 3 Institute of Epidemiology, Helmholtz Zentrum München, German Research Center for Environmental Health, Neuherberg, Germany; 4 Department of Neurology, BG Trauma Center, Murnau, Germany; 5 Global Public Health, Jeffrey Cheah School of Medicine and Health Sciences, Monash University Malaysia, Bandar Sunway, Malaysia

**Keywords:** Psychology, Medical research, Risk factors

## Abstract

Gender specific all-cause mortality risk associated with a *high* somatic symptom burden (SSB) in a population-based cohort was investigated. The study population included 5679 women and 5861 men aged 25–74 years from the population-based MONICA/KORA Cohort. SSB was assessed following the Somatic Symptom Scale-8 and categorized as *very high* (≥ 95th percentile), *high* (60–95th percentile), *moderate* (30–60th percentile), and *low* (≤ 30th percentile). The impact of SSB on all-cause mortality risk within a mean follow-up period of 22.6 years (SD 7.1; 267,278 person years) was estimated by gender-specific Cox regression models adjusted for sociodemographic, lifestyle, somatic and psychosocial risk factors, as well as pre-existing medical conditions. Approximately 5.7% of men and 7.3% of women had *very high* SSB. During follow-up, 3638 (30.6%) mortality cases were observed. Men with a *very-high* SSB had 48% increased relative risk of mortality in comparison to men with a *low* SSB after adjustment for concurrent risk factors (1.48, 95% CI 1.20–1.81, *p* < .0001), corresponding to 2% increased risk of mortality for each 1-point increment in SSB (1.02; 95% CI 1.01–1.03; *p* = 0.03). In contrast, women with a *very high* SSB had a 22% lower risk of mortality (0.78, 95% CI 0.61–1.00, *p* = 0.05) and women with *high* SSB had an 18% lower risk of mortality (0.82; 95% CI 0.68–0.98, *p* = 0.03) following adjustment for concurrent risk factors. The current findings indicate that an increasing SSB is an independent risk factor for mortality in men but not in women, pointing in the direction of critical gender differences in the management of SSB, including women’s earlier health care utilization than men.

## Introduction

All individuals experience some extent of somatic symptoms during their lifetime, often manifesting as pain, fatigue, dizziness, shortness of breath, or perceived disturbances of organ functions. However, in up to 20% of the general population^1^ and a third of clinical populations^2^ somatic symptoms remain an ongoing source of distress^3^, particularly among women^4–7^. Despite the considerable prevalence of a high SSB, the nature and long-term outcomes of somatic symptoms remain poorly understood. Current explanatory models are integrative in nature, attributing perceived symptoms to an interplay of gender, socioeconomic, biological and psychological factors, as well as prior expectations of the individual^8^. So far, there is substantial evidence that SSB follows the the course of underlying diseases^9,10^ as well as deteriorating health associated with aging^4,6,7,9–14^.

Nevertheless, increasing evidence suggests that the severity of somatic symptoms can be applied as an independent measure of health in the general population^12^. According to a population-based review of 9 studies including 28,377 participants, a score of somatic symptom burden can predict general health status, disability and healthcare use beyond that of important confounders including depression, anxiety and the presence or absence of a medical condition^14^. Most importantly, this review further demonstrates that the effect of somatic symptom burden on health status or health care costs are similar whether the symptoms are driven by organic disease or are medically unexplained^15–19^, highlighting that it is the degree of somatic symptom burden that needs to be considered as an independent risk factor of ill health.

Early evidence from prospective studies indicate that a high somatic symptom burden is additionally predictive of mortality among community dwelling older adults^20,21^. In 1583 participants aged between 75 and 95 years, 28% of participants who had a high somatic symptom burden had 2.08 (1.49–2.90) higher odds of mortality during 5 years of follow-up, even following adjustment for age, gender, and medical comorbidities^20^. Similarly, in 3498 participants with a mean age of 69 years, the total physical symptom count was predictive of 1-year death even after controlling for clinical characteristics, chronic medical conditions, self-rated health, and affective symptoms (OR 1.10, 95% CI 1.07–1.13)^21^. However, as these studies are limited to older populations with relatively short follow-up periods, and do not include gender-specific differences, the effect of a high somatic symptom burden on the relative risk of mortality in men and women from the general population remains unknown.

In the current investigation, using a representative sample of participants from the general population, followed for a mean of approximately 22 years, we will assess whether somatic symptom burden is associated with an increased risk of all-cause mortality while considering a wide range of sociodemographic, lifestyle, somatic, and psychosocial confounders derived from a priori research on somatic symptom burden. Furthermore, it is well accepted in psychosomatic clinical settings that somatic symptoms have a substantial gender component which cannot be overlooked^22^—gender differences in somatic symptoms are so prominent that an eventual diagnosis of somatic symptom disorder is estimated to affect more women than men at a ratio of 10:1^23^. Hence, the ensuing analyses will be gender-specific to address the gender discrepancies in somatic symptom reporting^3–6^, namely, to examine whether the higher prevalence of increased somatic symptom burden in women leads to a higher risk for mortality in women in comparison to men.

## Methods

### Participants

The study population was taken from three independent baseline surveys including 13,426 participants aged between 25 and 74 years-old who participated in the Monitoring of Trends and Determinants in Cardiovascular Disease (MONICA)/Cooperative Health Research in the Region of Augsburg (KORA) cohort study. The three baseline surveys were conducted in 1984/1985, 1989/1990, and 1994/1995 as part of the multinational World Health Organization (WHO) MONICA project^24^. The follow-up mortality data was obtained from the KORA GEFU 4 study (Cooperative Health Research in the Region of Augsburg, Health Follow-up 4) in 2016. Detailed information on the study design, recruitment process and data collection of the KORA studies have been described elsewhere^25^. All procedures contributing to this work comply with the ethical standards of the relevant committees and comply with the Helsinki Declaration of 1975, as revised in 2013^26^. In the current analysis, missing follow up data (n = 102), and missing covariates at baseline (n = 1784) led to a pooled sample 11,540 participants. Drop out analyses revealed that participants with missing data were more likely to be older (*p* < 0.001), have higher levels of depression (*p* < 0.001).

### Socio-demographic and lifestyle factors

*Gender* (woman/man) was self-reported, without further differentiation of biological sex or gender identity. *Low educational level* was considered as having < 12 years of schooling^24^. *Employment* status was based on self-reported information from the participants^24^. *Physical activity* was considered as engaging in physical activity on average of ≥ 1 h/week throughout the year^27^. *Smoking* was based on current smoking of ≥ 1 cigarette/day^28^. *Alcohol consumption* was based on three categories including ‘none-low’, ‘moderate’ and ‘high’ (categorical men: 0 g/day, 0.1–39.9 g/day, ≥ 40 g/day; categorical women: 0 g/day, 0.1–19.9 g/day, ≥ 20 g/day)^29^. *Living arrangement* was assessed by whether the individual currently lives alone, irrespectively of the current relationship status^30^.

### Somatic factors

Blood pressure was measured on the right arm in a sitting position using a Hawksley random-zero sphygmomanometer, and three were taken half an hour after the clinical interview in 3-min intervals. Blood pressure was assessed by obtaining the average of the latter two repeated-blood pressure measurements, and *hypertension* was defined as ≥ 140/90 mmHg and/or use of antihypertensive medication^31^. Total cholesterol (TC) and high-density lipoprotein cholesterol (HDL-C) were measured as mg/dL in serum by enzymatic methods (CHOD-PAP, Boehringer Mannheim, Germany) and *dyslipidemia* was defined as the ratio of total cholesterol to high-density lipoprotein cholesterol ≥ 5.0^32^. *Body mass index (BMI)* was calculated as weight in kilograms divided by height in meters squared and *obesity* was defined as having a BMI ≥ 30 kg/m^2^ ^33^.

### Assessment of pre-existing medical conditions and recent health care use

The participants were asked for the following *pre-existing medical* *conditions*, diagnosed and treated by a physician within the last 12 months: any form of cancer, cardiac insufficiency, angina pectoris, coronary heart disease, myocardial infarction, stroke, circulatory disorder in arms or legs, thrombosis/phlebitis/varicosis, diabetes,hyperlipidemia/hypercholesterolemia, hyperuricemia/gout, metabolic syndrome, chronic bronchitis, lung/bronchial asthma, arthrosis, rheumatoid arthritis, gastrointestinal illness/ulcer, kidney disease, liver disease, goiter or another thyroid disease^12^. Participants were categorized accordingly into ‘no pre-existing medical conditions,’ ‘1 pre-existing *medical condition’* or ‘ ≥ 2 pre-existing conditions (*multimorbidity*)’. *Recent health care* was considered as having had a health care visit at least once within the last 4 weeks^12^.

### Psychosocial risk factors

*Depressed-mood* was assessed by the depression and exhaustion subscale (DEEX) of the von Zerssen symptom checklist^34^, whereby participants in the top tertile were considered to have ‘high depressed-mood’, participants in the middle tertile were considered as having ‘moderate depressed mood’ and participants in the lowest tertile were considered as having ‘no/low depressed mood’. *Social network* was assessed by the Berkman-Syme's Social Network Index, and the components of the index are weighted in an algorithm resulting in four categories^35^, whereby *social isolation* was defined as low intimate contacts—not married, fewer than six friends or relatives, and no membership in either church or community groups.

### Assessment of somatic symptom burden

*Somatic symptom burden* was assessed by creating a symptom severity score, based on to the previously established Somatic Symptom Scale-8 (SSS-8)^19^. Eight somatic symptoms comprising the same categories as the SSS-8 were derived from the von Zerssen symptom checklist^36^, including bowel pain, back pain, pain in the joints, headaches or pressure in the head, temporary shortness of breath, dizziness, feeling tired and insomnia^37^. Each item was measured on a four-point scale ranging from 0 (not present) to 3 (strong) leading to a somatic symptom score ranging from 0 to 24. The distribution was approximately normal and Cronbach’s α was estimated as 0.75 in the present study indicating a good reliability^37^. The somatic symptom burden score was analyzed as a continuous variable, and additionally categorized as *low* (up to 30th percentile), *moderate* (30–60th percentile), *high* (60–95th percentile) and *very high* (> 95th percentile), which is interpreted as the clinical cut-off in practice^19^.

### Follow-up and mortality endpoints

Death certificates were obtained from local health departments and coded for the underlying cause of death by trained personnel using the 9th revision of the International Classification of Diseases (ICD-9). In the mean 22.6-year follow-up (SD ± 7.1 years; max: 32 years; 267,278.508 person years), there were 3638 fatal events. For mortality analyses, event times were calculated as time to death. Subjects without events or with loss to follow-up were censored at the time point of the last follow-up^31^.

### Statistical analyses

Baseline characteristics of the study population were gender-stratified and according to the severity of somatic symptom burden. Significance of differences between groups were compared using Post Hoc Anova for continuous variables and Multivariate Logistic regression for categorical variables.

Gender stratified mortality rates were calculated according to the severity of somatic symptom burden using Poisson regression with offset^38^. Gender stratified Cox proportional-hazards models were computed to assess the association of somatic symptom burden with all-cause mortality in men and women, where low somatic symptom burden was considered as the reference group^39^. The confounding risk factors in the models were chosen based on a priori evidence of factors that are associated with SSB and fitted in a forward stepwise regression. Three multivariate models stratified by gender and adjusted for (1) sociodemographic (age, employment) and lifestyle factors (smoking, alcohol consumption, physical activity) (2) somatic factors (hypertension, hypercholesterolemia, recent health care, 1 pre-existing medical condition or multimorbidity) and (3) psychosocial factors (depressed mood, lives alone, social isolation) were calculated. Model 3 included all primary risk factors. All models included adjustment for ‘survey’, as there were 3 waves of data collection. The magnitude of confounding was computed by comparisons of the estimated measures of associations for the crude and each consecutive model. If the difference of between measures of association were more than 10%, then confounding was considered present. In order to assess potential protopathic bias, a time lag was introduced by excluding the outcome variable in the first year following data collection^40,41^. Sensitivity analyses included multiplicative interaction testing for modification with the risk factors in the fully adjusted model, first in the total population, followed by in men and women separately. Proportional hazards could be estimated by fitting models stratified by the risk factor categories and plotting the log–log survival curves for each risk factor, which were assessed for parallelism by visual inspection and statistical testing of the Schoenfeld residuals^42,43^. As deviations from parallelism were not observed for any SSB categories and the global *p*-values for Schoenfeld residuals did not reach statistical significance in any models, proportional hazards were assumed^44^. Sensitivity analyses included assessment of modification by all risk factors in the total sample using multiplicative interaction in Model 3.

A *p* value < 0.05 was considered as statistically significant for main analyses and interactions. All statistical evaluations were performed using SAS 9.4. The analysis and the description in this manuscript follow the STROBE guidelines for cohort studies^45^.

### Ethical standards

The authors assert that all procedures contributing to this work comply with the ethical standards of the relevant national and institutional committees on human experimentation and with the Helsinki Declaration of 1975, as revised in 2008.

## Results

**Figure 1:**
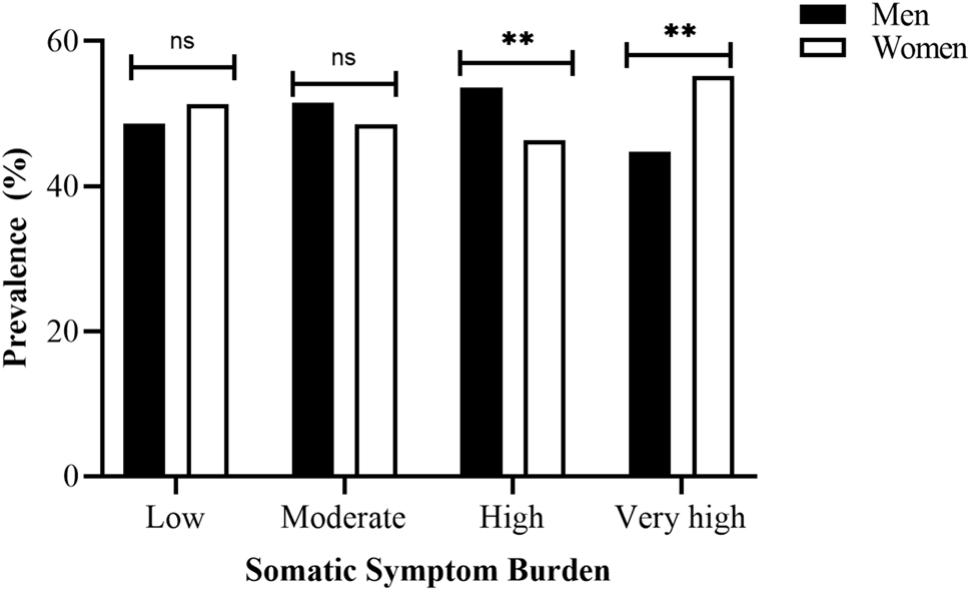
Prevalence of somatic symptom burden reported in men (n = 5861) and women (n = 5679) of the MONICA/KORA population-based cohort (N = 11,540).

The present data were derived from a total of 5679 women and 5861 men with a mean age of 46.6 (SD 13.2) and 47.8 (SD 13.5) years, respectively. As shown in Fig. 1, men had a higher prevalence of a *high* somatic symptom burden (29.0% vs. 25.9%), whereas women had a higher prevalence of a *very high* (7.3% vs. 5.7%) somatic symptom burden (test for trend *p* < 0.0001).

The baseline characteristics presented in Table 1 revealed a dose–response relationship between the severity of somatic symptom burden and increasing concurrent risk factors in both men and women—participants with an increasing severity of somatic burden were older, unemployed, or retired, less educated, physically inactive, more frequently had hypertension, hypercholesterolaemia, pre-existing medical conditions, and more frequent visits to a physician in comparison to participants with a low somatic symptom burden. The exception to this trend was for smoking and alcohol consumption, which decreased with increasing somatic symptom burden. Furthermore, a significant difference between men and women was found for the use of recent health care—women with *high* somatic symptoms were more likely than men to have had used recent health care, whereas men with *very high* symptoms had used more recent health care than women.

**Table 1:** Baseline characteristics of women (n = 5679) and men (n = 5861) according to somatic symptom burden in the MONICA/KORA population (N = 11,540).

		Women				Men				
		Somatic symptom burden				Somatic symptom burden				
Baseline characteristics, *n (%)*	Total (n = 5679)	Low n = 1795	Moderaten = 1995	High n = 1474	Very high n = 415	Total n = 5861	Low n = 1701	Moderaten = 2118	High n = 1705	Very high n = 337
**Sociodemographic**										
Age (mean, SD)	46.6 (13.2)	42.1 (12.6)	46.0 (12.9)***	50.8 (12.4)***	54.4 (11.4)***	47.8 (13.5)	43.7 (13.3)	46.4 (13.4)***	52.2 (12.4)***	56.2 (10.3)***
Low education	4489 (79.1)	1307 (72.8)	1555 (77.9)***	1251 (84.9)***	376 (90.6)***	3787 (64.6)	968 (56.9)	1305 (61.6)***	1241 (72.8)***	273 (81.0)***
Not employed	2997 (52.8)	883 (50.8)	1033 (51.8)	821 (55.7)***	260 (62.7)***	1598 (27.3)	336 (19.8)	486 (23.0)*	616 (36.1)***	160 (47.8)***
Retired	878 (15.5)	156 (8.7)	266 (13.3)***	328 (22.2)***	128 (30.8)	1311 (22.4)	250 (14.7)	386 (18.2)	528 (31.0)***	147 (43.6)***
**Lifestyle factors**										
Smoking	1249 (22.0)	433 (24.1)	439 (22.0)	294 (20.0)*	83 (20.0)*	1894 (32.2)	619 (36.4)	640 (30.2)*	520 (30.5)*	115 (34.1)
Alcohol consumption	1098 (19.3)	349 (19.4)	411 (20.6)	265 (18.0)*	73 (17.6)*	1886 (32.2)	556 (32.7)	672 (31.7)	569 (33.4)	89 (26.5)***
Physically inactive	2217 (39.1)	844 (47.1)	795 (40.0)***	469 (31.8)***	109 (26.3)***	2618 (44.7)	873 (51.4)	1038 (49.0)	609 (35.7)***	98 (29.1)***
**Somatic factors**										
BMI	26.0 (4.8)	25.3 (4.6)	25.8 (4.7)***	26.8 (4.8)***	27.3 (4.9)***	27.0 (3.6)	26.6 (3.5)	26.9 (3.5)*	27.4 (3.6)***	28.2 (3.8)***
Hypertension	1697 (29.9)	412 (22.9)	578 (29.0)***	525 (35.6)***	182 (43.9)***	2598 (44.3)	691 (40.6)	936 (44.2)***	779 (45.7)***	192 (57.0)***
Hypercholesterolemia	937 (16.9)	231 (13.2)	302 (15.6)*	309 (21.5)***	95 (23.5)***	2581 (44.7)	732 (43.6)	897 (43.0)	785 (46.7)	167 (50.4)***
**Pre-existing conditions**										
Medical condition^a^	1490 (26.2)	406 (22.6)	504 (25.3)***	605 (31.1)***	101 (29.2)***	1295 (22.1)	286 (16.8)	450 (21.2)***	471 (27.6)***	88 (26.1)***
Multimorbidity^b^	1020 (17.9)	115 (6.4)	302 (15.1)***	410 (27.8)***	193 (46.5)***	997 (17.0)	128 (7.5)	257 (12.1)***	447 (26.2)***	165 (49.0)***
Recent health care	2446 (43.2)	606 (33.8)	815 (41.0)***	775 (52.6)***	250 (60.4)***	2121 (36.2)	423 (24.9)	700 (33.1)***	778 (45.7)***	220 (65.5)***
**Psychosocial factors**										
Depressed mood	1379 (24.3)	77 (4.3)	349 (17.5)***	661 (44.8)***	292 (70.4)***	1423 (24.3)	66 (3.9)	336 (15.9)***	749 (43.9)***	272 (80.7)***
Socially isolated	829 (15.4)	208 (12.2)	267 (14.0)	271 (19.5)***	83 (22.6)***	597 (10.5)	175 (10.6)	201 (9.8)	192 (11.7)	29 (9.3)
Lives alone	1378 (24.3)	358 (19.9)	489 (24.5)***	399 (27.1)***	132 (31.8)***	1022 (17.4)	331 (19.5)	373 (17.6)	264 (15.5)*	54 (16.0)

^a^1 pre-existing medical condition.

^b^ ≥ 2 pre-existing conditions (multimorbidity).

*p < 0.05, ***p < 0.001.

As further shown in Table 1, there was a strong link between the severity of somatic symptom burden and psychosocial risk factors. Namely, up to 80% of men and 70% of women with a *very high* symptom burden had severe depressed-mood—in stark contrast to 4.3% of women and 3.9% of men with a *low* symptom burden. However, gender differences were found in the associations between social relationships and somatic symptom burden, whereby women who lived alone and/or were socially isolated had a substantially higher somatic symptom burden, but men did not experience an association between their social relationships and somatic symptom burden.

### All-cause mortality risk

**Figure 2:**
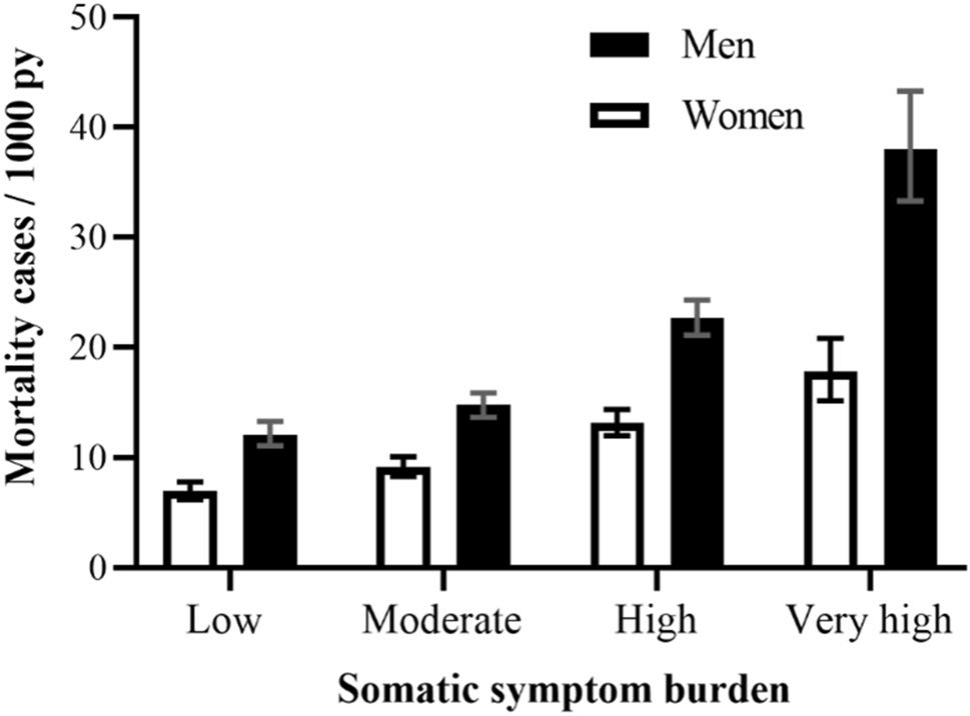
Absolute rate of all-cause mortality per 1000 person years (95% CI) according to somatic symptom burden in women (n = 5679) and men (n = 5861) of the MONICA/KORA population-based cohort (N = 11,540).

During a mean follow-up period of 22.6 years (SD 7.1), 3531 (30.6%) mortality cases were observed. As displayed by the survey-adjusted absolute mortality rates in Fig. 2, men, and women with increasing SSB levels experienced higher mortality rates / 1000 person-years in comparison to those with low SSB, but statistical significance was only reached in men (*p* < 0.0001) and not in women (*p* = 0.21).

Nevertheless, following adjustment for concurrent risk factors, the relative risk of all-cause mortality according to somatic symptom burden led to opposing yet significant results for men and women (Table 2). Men who reported a *very high* somatic symptom burden had a 48% higher risk of mortality than men with a *low* somatic symptom burden that remained significant even after adjustment for sociodemographic, lifestyle, somatic, and psychosocial risk factors, as well as pre-existing medical conditions (HR 1.48; 95% CI 1.12–1.81, *p* = 0.0003). Likewise, using the continuous somatic symptom burden score, the fully adjusted model showed that men had a 2% increased risk of mortality for each 1-point increment in somatic symptom burden (HR 1.02; 95% CI 1.01–1.03; *p* = 0.03). In contrast, women who presented a *high* somatic symptom had an 18% lower risk of mortality (HR 0.82; 95% CI 0.68–0.98, *p* = 0.03), and women with a *very high* somatic symptom burden had a 22% lower risk or mortality (HR 0.78; 95% CI 0.61–1.00, *p* = 0.05) in comparison to women with a low somatic symptom burden. The lower risk of all-cause mortality in women with an increasing somatic symptom burden was also evident when using the continuous somatic symptom score in the fully adjusted model, although statistical significance was not reached (HR 0.99; 95% CI 0.97–1.00, *p* = 0.18).

**Table 2:** Adjusted Hazard ratios (95% CI) of all-cause mortality in women (n = 5679) and men (n = 5861) according to somatic symptom burden in reference to participants with a ‘low’ somatic symptom burden, and the effect of concurrent risk factors within this association in the MONICA/KORA cohort (N = 11.540).

	Crude Model		Model 1		Model 2		Model 3	
	Women	Men	Women	Men	Women	Men	Women	Men
**Somatic symptom burden**								
Moderate	1.32 (1.14–1.53)***	1.23 (1.10–1.39)***	**0.97 (0.84–1.13)**	**1.07 (0.5–1.20)**	**0.92 (0.79–1.07)**	**1.05 (0.93–1.18)**	**0.92 (0.78–1.08)**	**1.07 (0.94–1.22)**
High	1.95 (1.68–2.25)***	1.96 (1.75–2.19)***	**0.97 (0.83–1.12)**	**1.06 (0.94–1.19)**	**0.86 (0.74–1.01)**	**1.03 (0.91–1.16)**	0.82 (0.68–0.98)*	**1.06 (0.92–1.23)**
Very high	2.65 (2.20–3.20)***	3.45 (2.94–4.04)***	**0.99 (0.82–1.21)**	1.61 (1.37–1.89)***	**0.85 (0.69–1.03)**	1.39 (1.17–1.65)***	0.78 (0.61–1.00)*	1.48 (1.20–1.81)***
**Sociodemograpic**								
Age			1.12 (1.11–1.13)***	1.10 (1.09–1.11)***	1.12 (1.11–1.12)***	1.11 (1.06–1.12)***	1.09 (1.10–1.12)***	1.10 (1.09–1.10)***
Low education			1.12 (0.95–1.31)	1.12 (1.01–1.23 )*	1.07 (0.90–1.27)	1.06 (0.89–1.25)	1.06 (0.89–1.26)	1.10 (0.99–1.22)
Not employed			1.23 (1.06–1.42)**	1.98 (1.80–2.18)***	1.17 (1.01–1.36)*	1.36 (1.21–1.54)***	1.22 (1.04–1.42)*	1.35 (1.20–1.52)***
**Lifestyle factors**								
Smoking			1.75 (1.52–2.03)***	1.99 (1.80–2.18)***	1.83 (1.58–2.12)***	2.07 (1.88–2.28)***	1.80 (1.55–2.10)***	1.99 (1.80–2.19)***
Physical inactivity			1.26 (1.12–1.43)***	1.23 (1.13–1.35)***	1.23 (1.09–1.40)**	1.21 (1.11–1.33)***	1.17 (1.02–1.34)**	1.20 (1.09–1.32)***
Alcohol consumption			0.87 (0.75–1.01)	1.04 (0.92–1.17)	0.94 (0.80–1.09)	1.05 (0.92–1.18)	0.96 (0.82–1.13)	1.06 (0.94–1.20)
**Somatic factors**								
BMI					1.02 (1.00–1.03)***	1.02 (1.00–1.03)**	1.02 (1.01–1.04)***	1.02 (1.00–1.03)**
Hypertension					1.30 (1.16–1.47)***	1.41 (1.29–1.55)***	1.35 (1.19–1.54)***	1.44 (1.31–1.59)***
**Pre-existing conditions**								
Medical condition^a^					1.09 (0.95–1.25)	1.24 (1.11–1.37)***	1.09 (0.95–1.26 )	1.22 (1.10–1.36)***
Multimorbidity^b^					1.30 (1.13–1.50)***	1.29 (1.11–1.37)***	1.32 (1.14–1.53)***	1.27 (1.13–1.42)***
Recent health care					1.24 (1.11–1.39)***	1.14 (1.04–1.25)**	1.22 (1.14–1.53)***	1.15 (1.05–1.27)**
**Psychosocial factors**								
Depressed mood							1.04 (0.94–1.15)	0.96 (0.80–1.04)
Lives alone							1.19 (1.04–1.36)*	1.43 (1.25–1.63)***
Social isolation							1.31 (1.04–1.67)*	1.26 (1.06–1.51)**

^a^1 pre-existing medical condition’.

^b^ ≥ 2 pre-existing conditions (multimorbidity).

*p < 0.05, **p < 0.01, ***p < 0.001.

Values in [bold] indicate the exposure variable.

The magnitude of confounding in the somatic symptom burden and all-cause mortality link showed significant confounding effects by concurrent risk factors (Table 3). Socioeconomic factors including age, education and employment had the largest confounding effect, substantially decreasing the crude all-cause mortality risk of SSB in men and women. The lowest level of confounding was by concurrent psychosocial risk factors, including depressed mood, living alone and social isolation, whereby men had a negative magnitude of confounding, indicating that the association is underestimated in men.

**Table 3:** Magnitude of Confounding (%) in the stepwise adjusted cox regression models estimating the association between somatic symptom burden and all-cause mortality in women (n = 5679) and men (n = 5861) in Table 2.

	Crude model versus model 1		Model 1 versus model 2		Model 2 versus model 3	
	Women (%)	Men (%)	Women (%)	Men (%)	Women (%)	Men (%)
**Somatic symptom burden**						
Moderate	36.08	14.95	5.43	1.90	0	− 1.87
High	101.03	84.91	12.79	2.91	4.88	− 2.83
Very high	167.68	114.29	16.47	15.83	8.97	− 6.08

Lastly, although multimorbidity as a concurrent risk factor was substantially significant within the somatic symptom burden and mortality link for women (1.32, 95% CI 1.14–1.53, *p* < 0.0001) and men (1.27, 95% CI 1.13–1.42, *p* < 0.0001), there was no statistical interaction between pre-existing medical conditions and severity of somatic symptom burden (women *p* = 0.17, men *p* = 0.37), indicating that pre-existing medical conditions does not modify the effect of somatic symptom burden on mortality.

Introduction of a time lag excluding mortality in the first year after data collection (n = 60), attenuated the long-term relative risk between SSB and all-cause mortality to non-significance in women with a *very high* SSB (Model 3: HR 0.79, 95% CI 0.62–1.01, *p* = 0.06) but remained unchanged in women with a *high* SSB (Model 3: HR 0.82; 95% CI 0.68–0.99; *p* = 0.03). Furthermore, the significant relative risk of all-cause mortality in men with a *very high SSB* did not considerably differ following the introduction of a time lag (Model 3: HR 1.46; 1.19–1.79, *p* = 0.0003).

### Sensitivity analyses

The modification effect by each concurrent risk factor was computed in the association between somatic symptom burden and all-cause mortality (Model 3). Gender (*p* < 0.0001) was the only significant modified in the total sample. Gender specific analyses revealed that depressed-mood (*p* < 0.0001) and physical inactivity (*p* = 0.05) were significant effect modifiers for men, whereas no significant modifiers were observed for women.

## Discussion

In the present investigation including 5679 women and 5861 men from the general population, 29.1% of men and 25.9% women reported *high*, while 5.7% of men and 7.3% of women reported *very high* somatic symptoms. Although both men and women with increasing somatic symptom burden suffered from psychosocial and somatic risk factors^7,14^, men generally had worse health, but women were more likely to report a ‘*very high’* somatic symptom burden^3–6^. Despite this, women with a ‘*high’* somatic symptom burden were more likely to have received recent health care, whereas men were more likely to wait until their symptoms advanced to ‘*very high’.* Correspondingly, *very high* somatic symptom burden was independently associated a 48% increased risk of mortality in men during a mean of 22.6 years of follow-up, even following adjustment confounding risk factors, and significant modification by depressed-mood and physical inactivity. In contrast, somatic symptom burden had a protective role against mortality in women—the relative risk of mortality was 18% lower for women with *high* and 22% lower for women with *very high* symptom burden following adjustment, potentially due to their readiness to seek health care earlier than men^46^.

The present findings confirmed and extended the prospective association between somatic symptom burden and all-cause mortality in an existing study. Namely, in the MONICA/KORA population-based study including 11,895 participants aged 24–75 years-old and followed for 12 years, e*xcessive symptom reporting* (ESR) in men with no chronic diseases demonstrated a measurable increased survival benefit compared to chronic disease participants with high symptom reporting (HR 0.68; 95% CI 0.48–0.97), however, in women, the most favorable outcome emerged in the population with high symptom reporting and no chronic disease (HR 0.68; 95% CI 0.36–1.31; *P* = 0.25)^12^. This finding, although not statistically significant, was attributed to *‘greater female-specific awareness of interoceptive cues and readiness to seek medical help may have led to more prevention-oriented behavior in women’*^12^, comparably to the current findings. However, we have found further evidence that somatic symptom burden may have a significant protective effect against mortality in women when concurrent risk factors are considered, and an independent risk factor mortality in men, even in the presence or absence of pre-existing medical conditions. Specifically, the magnitude of confounding revealed that the most significant risk factors to consider are socioeconomic factors including age, education, and employment status. Although additional studies were also in line with our findings for the somatic symptom burden and mortality link in men^15,21^, these studies were not gender-specific and included a substantially older study population, hence direct comparison to the current study is not feasible.

The prospective findings herein can be elucidated in light of the male–female health survival paradox^47^, that is, although females are more often ill^48,49^ they tend to have a longer life expectancy than males^47^. The underlying reasons for this paradox are not fully understood, but ‘feminine gender characteristics’^50^, including women’s processing and handling of somatic symptoms by readiness to seek medical help may contribute^46^. In addition, neurobiological mechanisms involving the female sex hormone, estrogen, are thought to increase the pain sensation in women, partially due to presence of estrogen receptors on nerve cells^51–54^. In turn, the amplification of pain mechanisms can activate an inflammatory response^55^, inducing more ‘sickness behaviour’^56,57^. However, emerging findings show that estrogen may also down regulate systematic inflammation—leading to an accelerated and more efficient inflammatory (and perhaps secondary anti-inflammatory) response compared with males^58^. Essentially, although females may experience somatic symptoms before males^22^, they may also develop protective mechanisms sooner and more effectively. Hence, the current findings suggest that somatic symptoms may indirectly increase women’s lifespan by adaptive behavioural or biological mechanisms, although these longer years may not necessarily be healthy years^49,59^.

The limitation of the current observational study is that direct cause and effect conclusions between somatic symptom burden and mortality cannot be discerned. Drop out analyses revealed that excluded participants with missing data were more likely to be older and have higher depressed mood in comparison to the current sample, hence our results might have underestimated the effect of SSB on all-cause mortality. Furthermore, although we have adjusted for a comprehensive set of confounding variables, we cannot exclude that risk factors not included herein may have biased the current results. For instance, the contrasting association between SSB and mortality in men and women may be linked to different comorbidity patterns and differences in severity of disease in men and women. Even though we have adjusted our analyses for somatic factors (hypertension, hypercholesterolemia and multimorbidity), other diseases/comorbidities and/or more severe disease in men that were not specifically included into the analysis might have an influence on all-cause mortality^60^. The current analyses did not indicate a substantial protopathic bias, however, the protective association between a *very high* SSB and all-cause mortality risk in women was attenuated to non-significance (*p* = 0.06) following the introduction of a time lag. This finding suggests that additional measures taken by women with a *very high* SSB (e.g., increased use of mental health services in women^61^) could have led to reverse causality. However, the heterogeneity of a large sample of participants randomly drawn from the population, the comprehensive set of variables, and the long follow-up period is expected to increase the validity of the current study.

In conclusion, the current findings have provided a real-world perspective of men and women suffering from somatic symptom burden in the community. An increasing somatic symptom burden, assessed by a brief somatic symptom inventory, has confirmed the exceeding concurrent cluster of risk factors in men and women^14^. Furthermore, following adjustment for these risk factors, somatic symptom burden was associated with an increased risk of mortality in men, yet had a protective role against mortality in women, potentially due to women’s sooner use of health care. Hence, efforts to improve somatic symptom burden at a population level must begin in primary care settings by increasing effective communication and encouragement for patients to overcome ‘white-coat silence’ and voice their concerns, with a particular focus on men^62^. For instance, public health initiatives aiming to improve men’s negative attitudes towards seeking health care such as ‘MENtion it to a doctor’ campaign by the Cleveland Clinic are endorsed^63^. Lastly, with respect to therapeutic interventions in managing somatic symptom burden^3^, a multidisciplinary health care approach that effectively addresses the exceedingly high comorbidity between somatic symptom burden, depressed-mood^64^ and unhealthy behaviors are recommended.

## Data Availability

The data could be requested from the MONICA/KORA-Myocardial Infarction Registry Augsburg, Germany.
