# Author Correction: Gender specific somatic symptom burden and mortality risk in the general population

**DOI:** 10.1038/s41598-022-25157-7

**Published:** 2022-11-29

**Authors:** Seryan Atasoy, Constanze Hausteiner-Wiehle, Heribert Sattel, Hamimatunnisa Johar, Casper Roenneberg, Annette Peters, Karl-Heinz Ladwig, Peter Henningsen

**Affiliations:** 1 Department of Psychosomatic Medicine and Psychotherapy, Klinikum Rechts Der Isar, University Hospital Rechts Der Isar, Technische Universität München, Langerstr. 3, 81676 Munich, Germany; 2 Department of Psychosomatic Medicine and Psychotherapy, University of Giessen and Marburg, Marburg, Germany; 3 Institute of Epidemiology, Helmholtz Zentrum München, German Research Center for Environmental Health, Neuherberg, Germany; 4 Department of Neurology, BG Trauma Center, Murnau, Germany; 5 Global Public Health, Jeffrey Cheah School of Medicine and Health Sciences, Monash University Malaysia, Bandar Sunway, Malaysia

Correction to: *Scientific Reports* 10.1038/s41598-022-18814-4, published online 05 September 2022

The original version of this Article contained an error in the spelling of the author Hamimatunnisa Johar, which was incorrectly given as Hamimatunissa Johar.

The original Article has been corrected.

